# Spinal Muscular Atrophy Scoliosis in the Era of Background Therapies—A Review of the Literature

**DOI:** 10.3390/jcm13123467

**Published:** 2024-06-14

**Authors:** Fred Ruythooren, Pierre Moens

**Affiliations:** 1 Department of Orthopaedic Surgery, University Hospitals Leuven-Gasthuisberg, 3000 Leuven, Belgium; 2 Institute for Orthopaedic Research and Training (IORT), University Hospitals Leuven-Gasthuisberg, 3000 Leuven, Belgium

**Keywords:** spinal muscular atrophy, SMA, spinal deformities, scoliosis

## Abstract

Spinal deformities are considered an important complication of neuromuscular disorders such as spinal muscular atrophy (SMA). SMA patients typically develop progressive early-onset scoliosis, which is associated with increased functional decline, discomfort, and respiratory dysfunction. Over the second decade of the twenty-first century, a lot has changed in terms of the therapeutic options available to people with SMA. Specifically, the use of pharmaceutical agents such as nusinersen (Spinraza), onasemnogene abeparvovec (Zolgensma), and risdiplam (Evrysdi) has dramatically changed the landscape for SMA patients. These medications significantly alter motor- and respiratory functioning, as well as the natural progression of spinal deformities. When evaluating these agents and their impact on the development of scoliosis and motor functioning, it is important to consider the timing of treatment initiation. In patients treated after they had already developed symptoms, a shift of phenotype to a less severe subtype has been observed. This results in a delay in the onset of scoliosis for the less severe SMA types and an increase in early-onset scoliosis for the severe types in patients who would typically not live to develop scoliosis. Patients who receive treatment before they develop symptoms achieve almost normal motor functioning and will likely have a significant decrease in scoliosis prevalence or at least delay its onset.

## 1. Introduction

The term “neuromuscular scoliosis” describes a variety of pathologies with different evolutions and severities, sometimes even within the same condition. Management can differ greatly, and generalization is not acceptable. Spinal muscle atrophy (SMA) is a good example of this phenotypic variability.

Spinal muscular atrophy is a group of recessive and progressive neuromuscular diseases defined by abnormalities of the lower motor neurons of the spinal cord, leading to the weakness and atrophy of voluntary muscles, caused by biallelic mutations of the *SMN1* gene. The severity of SMA is inversely related to the quantity of copies of the *SMN1* gene’s centromeric form (*SMN2*). The incidence of SMA has been estimated at between 7.8 and 10 per 100,000 live births [1].

Historically, SMA has been classified into four clinical subtypes (I, II, III, IV) according to age of onset and the motor milestones achieved. SMA type I is the most severe form of the condition. It is characterized by muscle weakness which can be present at birth or emerge within the first 6 months of life. These children are unable to control their head movements or sit without support (“non-sitters”); their chances of survival beyond early childhood are slim [1,2]. SMA type II represents a moderate severity of the disease, with muscle weakness emerging in children aged 6 to 18 months. They may initially sit without assistance (“sitters”); some may even stand or walk with assistance. Progressing disability means they will lose some of these motor functions over time. Life expectancy for type II SMA varies, but these individuals will most live until early adulthood [1,2]. SMA type III is a milder form of the condition; symptoms start developing after the age of 18 months. They have the ability to learn to walk and stand without assistance (“walkers”); however, these activities may become more difficult over time [1,2]. SMA type IV is the mildest form of the condition, and the first symptoms occur in early adulthood. Symptoms include muscle weakness, tremors, and minor respiratory issues. Individuals with SMA type III and IV typically have a normal life expectancy [3,4]. An overview of these clinical subtypes can be found in Table 1.

In this literature review, we will address recent developments in therapeutic options for SMA, and particularly its influence on scoliosis management. Over the last ten years, the possibilities and therapeutic options for SMA patients have changed significantly with the introduction of nusinersen (Spinraza, FDA approved in December 2016), onasemnogene abeparvovec (Zolgensma, FDA approved in May 2019), and risdiplam (Evrysdi, FDA approved in August 2020).

A comprehensive search of the literature was conducted, utilizing databases including PubMed, Embase, Web of Science, Medline, and the Cochrane Library, covering the period from 2016 to March 2024.

## 2. Background

The development of spinal deformities in SMA and other neuromuscular disorders results from the weakness of axial muscles and failure to sufficiently support the growing spine. Spinal deformities are seen as a significant complication of these disorders [5]. SMA patients typically have a hypotonic spinal curve, which can result in progressive and severe scoliosis at an early age, particularly in SMA type I and II [5,6]. Severe scoliosis may significantly impact motor functioning and will negatively impact respiratory functions. A large percentage of SMA patients develop scoliosis, with onset and severity typically associated with the severity of the SMA subtype [6,7].

SMA is most often associated with a C-shaped curvature of the thoracolumbar spine or, less commonly, an S-shaped curve, often accompanied by pelvic obliquity and increased thoracic kyphosis [6,7,8]. The resulting changes can severely affect sitting ability and respiratory function, due to both the altered shape of the trunk and diminished rib movement [8].

The natural development of scoliosis in SMA shows a gradual worsening of spinal curvature initially, with a progression rate of 7.2 degrees (cobb angle) per year. This is followed by a period of rapid progression of 10.2 degrees per year in the 18 months prior to surgery [6]. This progression occurs more slowly in less severe subtypes of SMA [6,7].

The likelihood of needing scoliosis surgery is approximately 80% for individuals with SMA types Ic and II [6,7]. According to Wijngaarde et al., type IIa patients, who only acquired the ability to sit, underwent surgery at a significantly younger age compared to those in the type IIb category, who learned to both sit and stand [7]. In contrast, only 40% of those with SMA type IIIa may require surgical intervention, with the necessity for surgery closely linked to the age at which ambulation is lost; earlier loss of mobility, particularly before the age of 10, predicts a poorer prognosis, with surgery needed in up to 70% of these cases [6,7]. According to a study by Ribero et al., the median age of loss of ambulation in SMA type IIIa individuals is 13.4 years compared to 44.2 years in SMA type IIIb individuals [6]. Accordingly, the necessity of scoliosis surgery in SMA type IIIb and IV individuals is low (2–5%) [6].

The median age for scoliosis surgery is around 10 years. The age at which scoliosis surgery is deemed necessary is associated with the subtypes, indicating that individuals with milder forms of SMA tend to undergo surgery later than those with more severe manifestations. Consequently, the decline in motor function is a determinant for both the necessity and timing of surgical intervention [7]. This underscores the importance of preserving and stabilizing motor functioning and development even into later years [6,7,8].

## 3. Treatment of Scoliosis

### 3.1. Non-Surgical Management

While bracing has been found to be ineffective in controlling spinal deformity in SMA, as highlighted by Granata et al., it still holds value in aiding positioning and enhancing the stability of the seated posture [9,10]. Nonetheless, it is important to acknowledge that bracing may negatively affect walking capabilities and respiratory function, as demonstrated in the research by Tangsrud et al. This underscores the complex balance between the benefits and drawbacks of non-surgical interventions in managing scoliosis in SMA patients [11].

### 3.2. Surgery

Guidelines recommend surgical intervention for skeletally immature patients with spinal curves exceeding 50 degrees. Surgery may also be necessary for patients who experience functional decline or difficulty sitting due to the deformity. In practice, this typically includes almost all patients with SMA type Ic and II.

Traditionally, early posterior spinal fusion (PSF) has been effective in correcting spinal deformities, as well as pelvic obliquity in neuromuscular scoliosis [12,13]. However, the suitability of this treatment is questionable for young SMA patients with early-onset scoliosis, especially types I and II, given its potential effects on their remaining growth, including diminished spinal growth, thoracic deviation potentially leading to reduced respiratory function, and progressive imbalances in spinal alignment between fused and non-fused segments. A study by Karol et al. revealed that early PSF does not effectively control spinal deformity, resulting in 24% to 39% of patients requiring surgical revisions. Additionally, restrictive pulmonary disease affected 43% to 64% of patients, and thoracic growth was reduced by 50%, which significantly contributed to major respiratory dysfunction [14].

An alternative to early PSF is fusionless scoliosis surgery (FSS), such as the use of growing rods (GRs), which allows for continued growth in height and helps prevent thoracic deviation, potentially allowing for increased lung volumes over time [15]. FSS has been demonstrated to be safe and effective in improving and maintaining spinal alignment in patients with SMA [16,17,18]. Miladi has developed a minimally invasive fixation technique that uses proximal and distal anchors connected by a bilateral double rod sliding construct. In cases of neuromuscular scoliosis, pelvic fixation is commonly achieved using iliosacral screws, which offer substantial stability as they cross three or four cortices [19]. Therefore, FSS not only minimizes invasiveness but also ensures a strong pelvic anchorage, which is necessary to address the commonly associated pelvic obliquity [20].

Consensus initiatives for best practice guidelines suggest that for SMA patients under 8 years old requiring surgery for spinal deformity (almost all SMA type I and II patients), surgeons should use growth-friendly instrumentation to preserve both growth potential and possibly pulmonary function [21]. For older patients and more skeletal mature patients, definitive PSF is effective at controlling curve progression without negatively impacting trunk height or pulmonary function [13].

The use of GR requires periodic surgeries under general anesthesia to manually adjust their length through lengthening procedures. The frequency of these procedures varies based on the individual’s growth rate. This approach does allow for progressive, gradual corrections of the spinal deformity, achieved through either symmetric or asymmetric lengthening of the rods [20,22].

Instrumentation with magnetic growing rods (MGRs), as discussed by Brooks and Sponseller, offers a non-invasive method for rod lengthening during growth using magnets, which has been demonstrated to be safe and effective in improving spinal deformities and pelvic obliquity [22,23,24,25]. However, the manufacturer advises that these rods be replaced every two years, and at the end of the growth phase, the MGR should be exchanged for conventional ones. This increases the frequency of anesthesia required. A recent study by Heiko et al. found that the lengthening potential in MGRs did not decrease over a four-year follow-up period when using a standardized lengthening protocol of 5 mm every three months, presenting a potential advantage over traditional GRs. With traditional GRs, the ‘law of diminishing returns’ often applies, where each subsequent lengthening results in less additional length [22].

The one-way self-expanding rod (OWSER) is a type of growing rod that passively extends in one direction as the spine grows. It allows for the progressive expansion of the rod by 1 mm per step, driven by natural growth. This eliminates the need for repeated surgeries or remote-controlled adjustments, such as those required with traditional GRs or MGRs. While the preliminary results are promising, additional research is required to fully assess its efficacy [26].

Common complications associated with growing rod systems include anchor pullout, rod breakage, and failure to lengthen. Complication rates for growing rod systems are reported to be around 30 to 40% [22,27]. One study suggests that FSS could result in significantly decreased vertebral bone mineral density (BMD) in SMA patients due to stress shielding, increasing the risk of implant-related complications [28]. While the literature is limited, these findings suggest that SMA patients could benefit from pharmaceutical therapy to increase BMD for a better surgical outcome and lower implant failure rates.

Definitive PSF at skeletal maturity is still the gold standard in children with neuromuscular scoliosis who have undergone FSS. However, according to recent studies, definitive fusion may not be necessary at the end of a lengthening program by FSS. A CT-guided study by Gaume et al. examined ten patients with neuromuscular scoliosis treated using GRs. It was observed that 93% of these patients experienced autofusion of the facet joints after reaching skeletal maturity, while also obtaining normal vertebral height [29]. Another study by Rewais et al. (2020) found similar results in a cohort of 12 non-ambulatory SMA patients treated with FSS [30].

The impact of surgical intervention on pulmonary function is still up for debate [5]. While scoliosis surgery is not associated with enhancing pulmonary function, it may contribute to a slower progression of pulmonary function decline [31]. Additionally, newer research indicates that FSS could be beneficial for pulmonary functioning by preserving spinal growth and thus improving thoracic morphology [16].

## 4. New Therapies

Over the second decade of the twenty-first century, a lot has changed in terms of the therapeutic options available to people with SMA. Specifically, the therapy of SMA now includes the use of three newly approved pharmacological agents. An overview of these new therapeutic options is shown in Table 2.

Nusinersen (Spinraza) is an intrathecally administered antisense oligonucleotide which received FDA approval in December 2016. It is designed to increase the inclusion of exon 7 in mRNA transcripts of the *SMN2* gene. As a result, there is a higher proportion of *SMN2* mRNA that includes exon 7, leading to the production of more functional, full-length *SMN2* protein [32,33,34]. Nusinersen is injected intrathecally on day 0, 14, 28, and 63, followed by a maintenance dose once every 4 months.

Onasemnogene abeparvovec (Zolgensma) is a gene therapy designed to introduce a fully functional human *SMN* gene into patients with SMA. Zolgensma received FDA approval in May 2019. Its working mechanism involves a one-time intravenous infusion using an adeno-associated virus serotype 9 as a delivery system. This virus carries a full-length human *SMN* cDNA controlled by a combined cytomegalovirus enhancer/chicken β-actin promoter, ensuring that the gene functions correctly. The structure of the AAV9 virus allows it to cross the blood–brain barrier (BBB), making it possible to administer the treatment intravenously. Once it is inside the central nervous system (CNS), the vector is endocytosed by cells, including motor neurons, where it transduces host cells to transcribe its double-stranded DNA [32,35,36].

Small molecules like risdiplam (Evrysdi) are *SMN2* splicing modifiers; they are designed to promote the inclusion of exon 7 in the *SMN2* mRNA transcript, resulting in the increased production of functional *SMN* protein. These compounds are taken orally once a day. Unlike nusinersen, which is also a splicing modulator, risdiplam influences the entire body and not only the CNS. Risdiplam received FDA approval in August 2020 [32,37,38,39].

When evaluating these therapeutic agents and their impact on the development of scoliosis and motor functioning, it is important to consider the timing of treatment initiation. The effectiveness of the treatment varies depending on whether it is initiated before or after symptoms appear, as well as the age of the patients and the number of *SMN2* copies [32]. It is known that presymptomatic-treated patients have significantly better outcomes compared to those who are treated after symptoms appear. This is thought to result from the process of nerve degeneration that occurs quickly within the first six months of life, highlighting the importance of preserving motor neurons before any clinical signs of deterioration become apparent to achieve better outcomes [32,40,41,42]. Short-term studies (5 years follow-up) indicate that treating patients before symptoms appear can lead to nearly normal motor function development [32,33,41,42]. These findings are consistent across all three different therapy types. The SPR1NT trial showed that presymptomatic treatment with onasemnogene abeparvovec gene therapy in patients with two *SMN2* copies at risk for SMA type I resulted in all patients gaining the ability to sit independently. Additionally, 11 out of 14 patients demonstrated normal motor function development at 18 months without requiring ventilatory support [40,42]. In patients with three *SMN2* copies, 14 out of 15 achieved normal motor function and could walk independently by 24 months [42]. Comparable results were seen in the NURTURE trial, which tested nusinersen for presymptomatic SMA treatment [33,34]. Preliminary results from the RAINBOWFISH trial for risdiplam also show promising outcomes [43]. In stark contrast, untreated SMA type I patients never develop the ability to sit independently, have a median ventilator-free survival of 8 to 10 months, and all either die or require permanent ventilatory support by the age of two [44]. While there is limited published data on the occurrence of spinal deformities in the presymptomatic treatment of SMA, it is generally assumed that there is a significant reduction in early-onset scoliosis. However, current follow-up periods extend only to about five years. It is anticipated that presymptomatic treatment will likely drastically reduce the incidence of scoliosis in SMA patients and/or delay the onset of spinal deformities. However, presymptomatic treatment relies on systematic newborn screening (NBS), as symptoms can begin around six months of age [3,4]. In the US, Canada, Taiwan, Japan, Russia, and several European countries, including Belgium, Norway, and Germany, newborn screening is already in place, as of December 2020, facilitating early diagnosis and intervention [32,45].

When treating SMA patients who are already symptomatic, treatment can alter their disease phenotypes. For instance, patients with SMA type I, who typically do not survive long-term, can survive and exhibit motor functions similar to those of SMA type II individuals when treated [32,35,38,46]. This shift in disease expression presents new clinical challenges. SMA type I patients, who ordinarily would not live long enough to develop scoliosis, now face a high risk of developing spinal deformities which may require surgical intervention as a result [47,48,49]. A study by Ip et al. observed that treating symptomatic pediatric SMA patients with nusinersen leads to increased scoliosis progression in SMA type I patients—individuals who would not have survived without treatment—while it decreases scoliosis progression in patients with SMA types II and III [50]. Phase III trials of symptomatically treated SMA type I patients have demonstrated an extended time to death or need for permanent ventilation compared to the sham control group. Those in the treated group exhibited markedly improved motor development, with some achieving the ability to sit upright [35,51]. Similar trials for SMA types II and III also reported significant motor development gains in comparison to control groups, further emphasizing the impact of symptomatic treatment on disease phenotypes [52,53]. Additionally, data suggest that the improvement in motor functioning among symptomatically treated patients correlates with the severity of scoliosis at the baseline. Patients with no or mild scoliosis tend to show greater improvements [49,54].

A recent Turkish retrospective study reported on scoliosis development and hip subluxation in 26 SMA type I patients receiving nusinersen. Ninety-three percent of these patients developed scoliosis before the age of two, with a mean Cobb angle of 39.3° ± 19.9°. Hip subluxation was observed in 16 patients (60.7%) [55]. The study does not clarify whether the patients had developed symptoms prior to starting treatment. However, since all patients began treatment before the introduction of newborn screening in Turkey in 2022, it is likely that they were already exhibiting symptoms when treatment commenced, again highlighting the shift in phenotype in symptomatically treated SMA type I patients who develop clinical features comparable to the traditional SMA type II phenotype.

The classification of SMA is evolving from traditional subtypes to a more detailed approach that considers the age of onset, number of *SMN2* copies, age at initiation of drug treatment, and current motor function levels (non-sitter, sitter, walker) to define clinical phenotypes. This allows for more precise monitoring of disease progression and enhances the tailoring of medical care [32,56].

## 5. Discussion

In this review article, we explore the advancements in surgical techniques for treating early-onset scoliosis in SMA patients, as well as the impact of background therapies on the development and progression of spinal deformities in this group. We have observed a significant shift in clinical phenotypes among symptomatically treated SMA type I patients who historically would not have survived. These patients now exhibit motor functions akin to those of SMA type II patients but still face a high risk of developing spinal deformities [32,35,40,50,51]. For SMA types II and III patients receiving symptomatic treatment, the progression of scoliosis appears to be slower compared to those in control groups [50]. In cases where treatment is administered in a presymptomatic stage, it could potentially prevent the development of scoliosis entirely or at least delay its onset, though longer follow-up is needed to confirm these possibilities. A shift in the classification of SMA from the traditional three subtypes to a more nuanced approach is likely inevitable. We have to take into account the age of onset, the number of *SMN2* copies—which also serve as a prognostic factor for treatment response—the age at initiation of drug treatment, and current motor function levels (non-sitter, sitter, walker) to more accurately define clinical phenotypes [40].

Thus, spinal deformities will remain in SMA patients and require surgical intervention [40]. In recent years, there has been a shift towards fusionless spinal surgery, which could overcome some of the pitfalls of traditional early posterior spinal fusion, such as diminished spinal growth, thoracic deviation potentially leading to reduced respiratory function, and progressive imbalances in spinal alignment between fused and non-fused segments [14,16]. Miladi introduced a minimal invasive technique for fusionless spinal surgery using a proximal and distal anchor connected by a bilateral double rod sliding construct [19]. Innovative instrumentation has been developed, including magnetic growing rods by Brooks and Sponseller, and the OWSER growing rod. These devices allow for progressive lengthening of the construct without the need for additional surgical interventions. The lengthening is achieved by either using remote-controlled magnets (MGRs) or passive progressive lengthening driven by natural growth (OWSER) [23,26]. Long-term follow-up data suggest that definitive spinal fusion at skeletal maturity may not be necessary as autofusion occurs at maturity in a very high percentage (93%) of patients [29,30].

All three background therapies for SMA differ in their routes of administration. Nusinersen requires intrathecal delivery every 4 months, which can be challenging in patients with severe spinal deformities or those who have undergone complex spinal surgeries. For instance, a study by Messina et al. highlighted that the injection procedure failed on the first attempt in 24 out of 120 patients due to significant spinal deformities [57]. To address this, some experts recommend the use of ultrasound-guided infiltrations, either interlaminar or transforaminal, which have shown high success rates when performed by experienced physicians [58,59,60]. This technique is preferred over CT or fluoroscopic-guided methods as it avoids repeated exposure to radiation. When surgical intervention for spinal deformity in patients is required, it is advisable to create a safe and reliable access point for intrathecal infiltration. This can be effectively achieved by performing a lumbar laminotomy or by creating an interlaminar lumbar fenestration at the convex side of the spine [61,62,63]. A study by Konigsberg et al. recommends using ‘skip’ constructs when considering traditional PSF. This involves leaving two or three levels, typically L2 and L3, untouched during surgery to facilitate easier access for intrathecal infusion [64]. Lastly, some authors describe the use of indwelling subcutaneous intrathecal catheters, which allows for the reliable outpatient administration of nusinersen [65].

One interesting issue with SMA therapies relates to their potential side effects on pregnancy, particularly as these treatments extend the lifespan of patients with severe SMA and enhance the lifestyle of those with less severe forms, potentially leading to more pregnancies among this patient group. Risdiplam has shown embryo-fetotoxic and teratogenic effects in animal studies, suggesting potential risks in human pregnancies [66]. While nusinersen is also not recommended during pregnancy, this is a precautionary measure as it has not yet been shown to cause reproductive toxicity in animals [67]. Given the reproductive risks associated with risdiplam, nusinersen might be the safer alternative for female patients considering pregnancy. Keeping in mind the potential complications in administering the drug intrathecally to those with complex spinal deformities or previous spinal surgeries, it may be advisable to always consider future possibilities of nusinersen treatment when planning surgical interventions for scoliosis in SMA patients by creating a safe access point for intrathecal infiltration.

Onasemnogene abeparvovec is a form of gene therapy which is administered through a single intravenous injection by slow infusion over about an hour. To be eligible for gene therapy, SMA patients must be tested for antibodies against the AAV9 viral vector used in the therapy. The presence of anti-AAV9 antibodies can inhibit vector transduction and reduce the therapeutic effect [68]. The long-term efficacy of onasemnogene abeparvovec is still under observation, and whether it will have a lifelong effect remains to be seen. Recent long-term follow-up studies have shown that the therapy continues to be effective up to 7.5 years post-administration [69]. The AAV vectors employed in gene therapy are replication-incompetent and remain episomal, which means that their expression can decrease as cells divide and die. However, since AAV is particularly effective in quiescent cell types such as neurons, which do not divide, onasemnogene abeparvovec has the potential to offer sustained benefits, potentially leading to lifelong effects [70]. If repeat administration of onasemnogene abeparvovec is necessary, the treatment may become ineffective due to an adaptive immune response to the viral vector [70,71].

The idea of combining therapies for SMA is emerging, although comprehensive data are still sparse. A study conducted in the United States using a mouse model with a severe form of SMA found that compared to administering a single treatment, the combination of nusinersen and risdiplam enhances *SMN* protein production. This dual approach not only improves motor function but also extends lifespan, showing effectiveness even after symptoms have manifested [72]. Another American study has reported potential beneficial effects in four patients treated with both onasemnogene abeparvovec and risdiplam [73]. Clinical trials are currently ongoing to evaluate the response to dual therapy combinations. Specifically, the RESPOND trial is testing the combination of nusinersen and onasemnogene abeparvovec in 60 infants, while the MANATEE trial is exploring the combination of risdiplam with an anti-myostatin agent, GYM329 (RO7204239), in 180 children [69,70].

All three new agents are designed to reverse degenerative processes, leading to improved functional outcomes. However, they do not remediate impairments caused by pre-existing neurodegeneration and muscle atrophy. A novel therapeutic approach involves targeting myostatin, a protein that negatively regulates muscle growth. Apitegromab, an investigational fully human monoclonal antibody, inhibits the activation of myostatin, theoretically promoting muscle growth and potentially reducing or even preventing muscle atrophy in SMA patients. The preliminary results from the TOPAZ phase II study of Apitegromab are promising [74,75].

## Figures and Tables

**Table 1:** Clinical subtypes of SMA.

SMA Subtype	Age of Onset	Maximum Motor Milestone	Life Expectancy
0/1A	Neonatal (0–1 months)	Nil	<6 months, often days–weeks
1B	<3 months	Unable to sit unsupported	<2 without respiratory support
1C	3–6 months		
II	6–18 months	Able to sit unsupported but unable to walk independently	20–40 years
IIIA	18–36 months	Able to walk independently	Almost normal
IIIB	>36 months		
IV	>21 years	Normal	Normal

Subtypes as described by Markowitz et al. (2012) [2].

**Table 2:** Overview of the therapeutic options for SMA.

Medication	Route of Administration	Working Mechanism	Date of FDA Approval
Nusinersen (Spinraza)	Intrathecally on day 0, 14, 28, and 63, followed by a maintenance dose once every 4 months.	Antisense oligonucleotide, which increases the inclusion of exon 7 in mRNA transcripts of the *SMN2* gene	December 2016
Onasemnogene abeparvovec (Zolgensma)	One-time intravenous infusion	Gene therapy designed to introduce a fully functional human *SMN* gene into patients with SMA	May 2019
Risdiplam (Evrysdi)	Orally once a day	*SMN2* splicing modifiers, designed to promote the inclusion of exon 7 in the *SMN2* mRNA transcript	August 2020
